# The Impact of Sleep Quality and Education Level on the Relationship between Depression and Suicidal Ideation in Parents of Adolescents

**DOI:** 10.3390/healthcare9091171

**Published:** 2021-09-06

**Authors:** Ji Yeon Shim, Sook Lee, Il Hyun Lee, Yoo Mi Jeong

**Affiliations:** 1 College of Nursing, Dankook University, 119 Dandae-ro, Dongnam-gu, Cheonan-si 31116, Korea; 12200392@dankook.ac.kr (J.Y.S.); moonlight@dankook.ac.kr (S.L.); 2 Knowledge Industry Center 174, StatEdu Institute of Statistics 514, Iksan-si 54630, Korea; dr.leeilhyun@gmail.com

**Keywords:** depression, suicide, school and education, child and adolescent, quantitative methodology

## Abstract

This study aimed to analyze the moderating effect of sleep quality and the moderated moderation effect of education level on the relationship between depression and suicidal ideations among middle-aged parents of adolescent children. This is a secondary analysis of a survey collected from a cross-sectional study. The inclusion criteria were middle-aged parents of adolescent children in D city, South Korea, who answered the survey questionnaires. A total of 178 completed questionnaires were used for the analysis. The moderating effect of sleep quality (B = −0.03, *p* = 0.736) and education level (B = −1.80, *p* = 0.029) on the relationship between depression and suicidal ideations was shown. It was confirmed that the moderating effect of sleep quality on the effect of depression on suicidal ideations differed according to the subject’s education level. The findings have implications for mental healthcare providers who can be educated on sleep hygiene based on the subject’s education level.

## 1. Introduction

**Figure 1:**
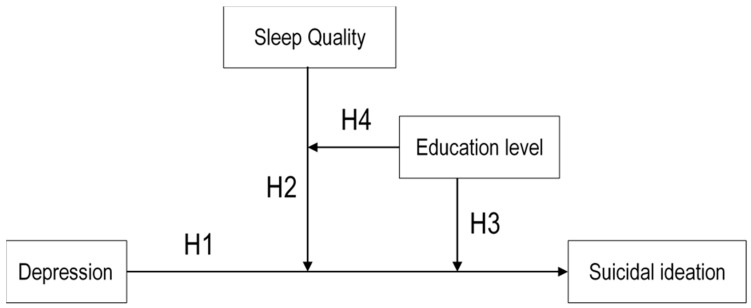
Conceptual framework. H, hypotheses.

Suicide is considered a worldwide problem affecting all age groups and is the 10th leading cause of death in the United States. It is the second-leading cause of death for those aged 10–34 years and the fourth-leading cause of death for those aged 35–54 years [1]. In South Korea, suicide is the second-leading cause of death in adults aged 40–50 years [2]. Most adults between 40 and 50 years face psychosocial development tasks related to generativity and stagnation while raising adolescent children [3] and are reported to be more psychologically and physiologically stressed, as compared to those in early adulthood [4]. Middle-aged parents with adolescent children feel more stress, emptiness, loneliness, and lower life satisfaction and fullness than middle-aged parents with children of any other age group [5]. Conflicts with children, paired with loneliness, affect depression in middle-aged parents [6]. Middle-aged parenting behaviors are directly or indirectly related to depression and suicidal ideations and influence the behaviors of adolescent children through their methods of communication and showing affection [7,8,9,10]. Therefore, to not only improve the mental health of middle-aged parents but also prevent suicide in their adolescent children, it is necessary to clarify the current status and mental health factors of middle-aged parents with adolescent children. Suicide occurs as an extension of suicidal ideations and suicidal motivations, and the suicide rate of adults with suicidal ideations is significantly higher than that of adults without suicidal ideations [11]. Life stress and depression are typical psychosocial factors affecting suicidal ideations in middle-aged adults [12]. People who experience depression are at risk of suicide and are repeatedly exposed to uncontrollable depression and suicidal ideations [13]. In middle-aged women, the incidence of depression is higher due to physiological changes and role conflicts, such as changes in female hormones during menopause, life stress, feelings of loss due to their child’s independence, and the increased burden of middle-aged parental support [14,15]. Middle-aged men complain of symptoms such as a decrease in male hormones, poor memory, muscle weakness, general weakness, and impotence due to male menopausal symptoms [13]. In summary, depression in middle-aged adults causes various mental and physical symptoms, along with low cognitive function, and this can lead to suicide if left untreated. The sleep quality can also affect suicidal ideations. The appropriate amount of sleep for adults is 7–9 h; when the sleeping time decreases to 7 h or less, the risk of depression and suicidal ideations increases [16]. The sleep time has a moderating effect on suicidal ideations. If the sleep time is short (1–5 h), the risk of suicidal ideations increases 1.33 times in the average adult, and the risk of suicidal ideations increases 1.92 times in adults experiencing depression, which implies that suicidal ideations tend to increase in sleep-deprived adults [17]. In the study of Basner et al. [18], which examined the sleep time of 30–50 year olds, the sleep time was the shortest in the middle-aged group compared to the other age groups and, also, in the case of middle-aged parents with children under the age of 18, with all the children attending school. Compared to middle-aged parents of children older than 18 years, the sleep time was shorter, and women were more affected [19]. A study by Owusu et al. [20] also reported that adults over 50 years old had a higher risk of suicidal ideations and characteristically more insomnia symptoms with poor sleep quality. The education level was one of the factors influencing sleep disorders. Compared to university graduates, those with a high school diploma or less were more likely to sleep for less than 6 h or more than 9 h of high-risk sleep [21]. Another study showed that higher educational attainment was associated with lower sleep duration [22] and also reduced the risk of suicide attempts [23]. Sleep disturbances also affect suicidal ideations, and the lower the education level of middle-aged and older adults, the higher the suicidal ideations [19,24]. As such, depression and suicidal ideations in middle-aged adults increase with poor sleep quality and lower education levels [16,17,20], and the lower the level of education, the poorer the sleep quality [22]. In addition, Sami et al. [25] reported that sleep problems have a moderating effect on depression, which mediates the effect of internet addiction on suicidal ideations in adolescents. However, studies have not focused on middle-aged parents with adolescents or how both qualities of sleep and education level affect depression and suicidal ideations. Therefore, it is necessary to determine the role the education level plays in the relationship between depression and suicidal ideations, as well as sleep quality. The purpose of this study was to examine how depression in middle-aged parents with adolescent children affects suicidal ideations and to verify the moderating effect according to middle-aged parents’ sleep quality and education level. Through the verification of such a model, we provide basic data for the development of a program for the prevention of suicidal ideations in a depressed group by revealing the role of the sleep quality and education level on the influence of depression in middle-aged parents with adolescent children on suicidal ideations. The hypotheses of this study are as follows (Figure 1).

**Hypothesis** **1** **(H1):** *Depression will have a direct effect on suicidal ideations.*

**Hypothesis** **2** **(H2):** *The sleep quality will have a moderating effect on the effect of depression on suicidal ideations.*

**Hypothesis** **3** **(H3):** *The education level will have a moderating effect on the effect of depression on suicidal ideations.*

**Hypothesis** **4** **(H4):** *The education level will have a moderated moderation effect on the sleep quality that moderates the effects of depression on suicidal ideations.*

## 2. Materials and Methods

### 2.1. Study Design and Sample

This secondary research study used a cross-sectional survey design. The inclusion criteria were middle-aged parents of adolescent children who were able to understand and answer the survey questionnaires. The number of samples for the multiple regression analysis, using the G-power 3.1 program, was calculated as approximately 160 samples (effect size = 0.15; α power = 0.85) [26]. Considering a dropout rate of 20%, the optimal sample size was 160–192. For this study, 178 of the 192 completed questionnaires were used for the analysis.

### 2.2. Data Collection and Ethical Consideration

This secondary study received an exemption from the Institutional Review Board of Dankook University (Approval No. 2020-05-022). The original study data were collected from two middle schools located in D City in South Korea between 2018 and 2019 using a self-administered questionnaire. The original data were collected after receiving official permission from both school principals and obtaining written informed consent from all the study participants. The consent form and questionnaires were distributed to the participants by each school nurse and school counselor at the two schools via mail. The participants were informed about the study’s purpose and provided with detailed instructions. An envelope with a stamp was given, and the parents who completed the survey immediately returned it by mail to the PI directly. The consent form included a statement about voluntary participation and protection of anonymity and confidentiality.

### 2.3. Measures

#### 2.3.1. Suicidal Ideations

The suicidal ideations questionnaire (SIQ) was developed by Reynolds [27] to measure current suicidal ideations and was adapted and revised into Korean by Shin [28]. The SIQ is based on a 6-point Likert scale with a total of 30 items, such as “I ideation it would be better if I was not alive”, “I ideation about committing suicide”, and “I ideation about how to commit suicide”. Each question could be answered as follows: 0 = none, 1 = last month, 2 = once a month, 3 = 2 to 3 times a month, 4 = once a week, 5 = 2 to 3 times a week, and 6 = almost every day. The total scores ranged from 0 to 180, and the higher the total score, the higher the number of suicidal ideations. The original SIQ was bought from Psychological Assessment Resource, Inc. (North Florida, FL, USA) [29], and the Korean version of the SIQ was developed by Shin [26]. Reynolds’ [27] Cronbach’s α was 0.97, Shin’s [28] Cronbach’s α was 0.96, and this study’s Cronbach’s α was 0.99.

#### 2.3.2. Depression

The Beck Depression Inventory-II (BDI-II) [30], which has a total of 21 items, was used and included statements such as “I do not feel sad”, “I am not particularly discouraged about the future”, and “I do not feel like a failure”. The responses were measured on a 4-point Likert scale (0 to 3 points), and the original scale’s Cronbach’s α was 0.92. In the United States, a BDI-II score of 0–13 points is indicative of minimal depression, 14–19 points indicates mild depression, 20–28 points indicates moderate depression, and 29–63 points indicates severe depression. The Korean version of the BDI-II was used in this study and was purchased from Korea Psychology [31]. The cut-off point was 14 points for mild levels of depression, as suggested by Psychological Assessment Resource, Inc. [32], and the Cronbach’s α was 0.80.

#### 2.3.3. Sleep Quality

Sleep quality was measured using the Korean version of the Pittsburgh Sleep Quality Index (PSQI-K) developed by Buysse et al. [33] and revised by Sohn et al. [34]. The PSQI is classified into seven component scores with a total of 19 items related to sleep duration and delay time related to sleep quality and the frequency and severity of specific sleep-related problems. Each item is rated on a 4-point Likert scale ranging from 0 to 3 points. The seven component scores were summed and presented as one score. The total PSQI scores range from 0 to 21 points, with 0 indicating no sleep problems and 21 indicating serious sleep problems. The higher the score, the lower the sleep quality [33]. At the time of development, the Cronbach’s α was 0.83, and the Korean version’s by Sohn et al. [34] was 0.84. The PSQI-K’s cut-off point was 8.5 points for distinguishing between poor and good sleepers [33]. In this study, the reliability was acceptable, and the Cronbach’s α was 0.7.

#### 2.3.4. Sample Characteristics

In this study, the education level refers to the education level of middle-aged parents with adolescent children and was classified as high school graduates or lower and university graduates or higher. The results of previous studies showed a significant difference in depression and suicidal ideations according to age, with depression increasing in middle-aged adults and older age groups [35]. As gender and age are among the demographic and sociological characteristics that are likely to affect this study, age was set as a control variable.

#### 2.3.5. Statistical Analysis

We analyzed the data using SPSS version 25.0 (IBM Corp., Armonk, NY, USA) and SPSS PROCESS macro version 3.4 [36]. The general characteristics of the subjects, suicidal ideations, sleep quality, and depression were based on descriptive statistics. A correlation analysis based on Pearson’s correlation coefficient was performed to examine the correlation between suicidal ideations, depression, sleep quality, and education level. Following the guidelines of Hayes [36], PROCESS macro model 3 was used to test moderated moderation effects. To confirm the moderation effects, the relationship between the direct effect of the predictor (H1: depression) and the indirect effect of the three moderators (H2: depression × sleep quality, H3: depression × education level, and H4: depression × sleep quality × education level) on suicidal ideations should be statistically significant. Bootstrapping was conducted to test the moderation model. An interaction effect was considered significant if its 95% bootstrap confidence interval from 10,000 bootstrap samples did not include the value 0.

## 3. Results

### 3.1. Sample Characteristics

Table 1 shows the demographic and social characteristics, such as gender, age, and education level, of the participants. Most participants were female (91.6%, *n* = 163), and the average age was 46.28 years (±3.21). In terms of education level, 64.4% of the participants graduated from high school.

### 3.2. Correlations between Depression, Sleep Quality, and Suicidal Ideations

Table 2 shows the correlations between depression, sleep quality, and suicidal ideations in middle-aged parents with adolescent children. The mean for depression was 6.34 (±6.45), the mean for suicide ideations was 8.13 (±19.56), and the mean for sleep quality was 4.24 (±2.57). Suicidal ideations were positively correlated with depression (*r* = 0.59; *p* < 0.001), sleep quality (*r* = 0.34; *p* < 0.001), and age (*r* = 0.15; *p* = 0.046). In addition, there was a positive relationship between depression and sleep quality (*r* = 0.37; *p* < 0.001).

### 3.3. Moderating Effects of Sleep Quality and Education Level on the Relationship between Depression and Suicidal Ideations

Table 3 summarizes the moderating effects of sleep quality and education level between depression and suicidal ideations when age and gender were controlled. Depression (B = 0.92, *p* < 0.001) and sleep quality (B = 0.87, *p* = 0.040) were significantly positively associated with suicidal ideations in Model 1. This model had 21% explanatory power to explain suicidal ideations, but sleep quality did not significantly affect suicidal ideations. Therefore, these results did not support H1. In Model 2, the hypothesis test for the moderating effects of sleep quality (B = 0.21, *p* = 0.003) and education level (B = 1.15, *p* = 0.002) on the relationship between depression and suicidal ideations were shown to be statistically significant. The explanatory power significantly increased by nine percentage points compared to Model 1 (*p* < 0.001), and these results supported H2 and H3 in this study. In Model 3, notably, the three-way interaction of depression, sleep quality, and education level was significant; the moderating effect of sleep quality on the relationship between depression and suicidal ideations was further moderated by the education level (B = 0.53, *p* < 0.001). The explanatory power significantly increased by six percentage points compared to Model 2 (*p* = 0.001), and these results supported H4 in this study.

**Figure 2:**
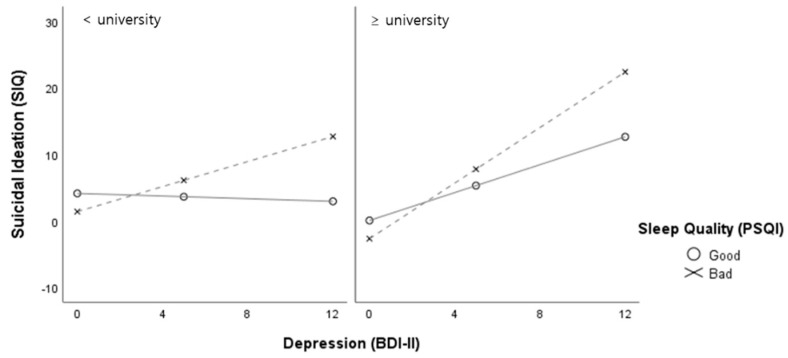
Slope test for the moderation effect of the sleep quality and education level.

**Figure 3:**
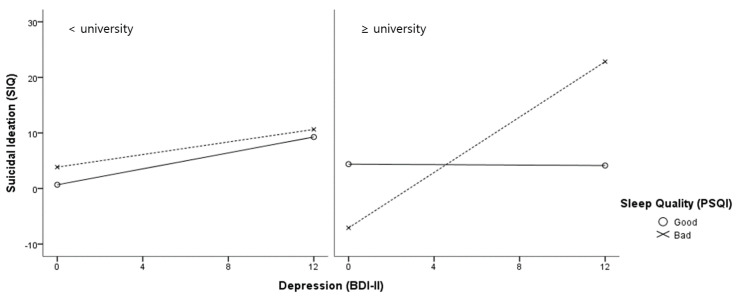
Slope test for the moderated moderation effect of the education level on depression, sleep quality, and suicidal ideations.

A simple slope effect test was conducted, as shown in Figure 2. In both the education level groups, depression significantly predicted the suicidal ideations with both good and poor sleep quality. Moreover, poor sleep quality showed a greater moderating effect on the relationship between depression and suicidal ideations than good sleep quality, regardless of the education level. A simple slope effect test of the moderated moderation effect of the education level showed that, in the university or higher graduation group, depression significantly predicted suicidal ideations (B = 0.51, *p* = 0.001), while, in the high school graduation group, the effect of depression on suicidal ideations was not statistically significant (see Figure 3). Significant moderated moderation effects of the education level on depression were observed only on the poor sleep quality in the university or higher graduation group.

**Figure 4:**
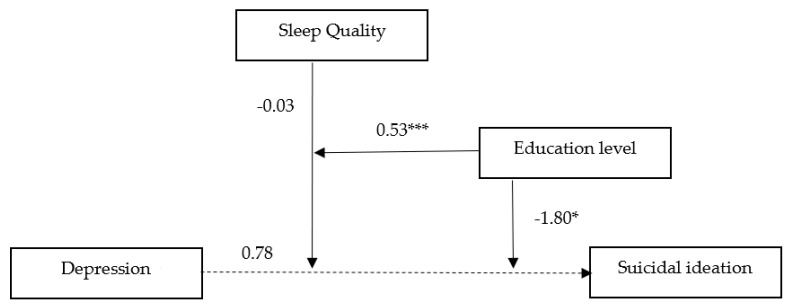
Moderated moderation effect of the sleep quality and education level between depression and suicidal ideations. An unstandardized regression coefficient (B) was reported as the moderation effect. * *p* < 0.05 and *** *p* < 0.001.

Figure 4 provides a visual summary of the results of the research model. Sleep quality has a moderating effect on the influence of depression on suicidal ideations, and the moderation effect of sleep quality is affected by the participant’s education level.

## 4. Discussion

The purpose of this study was to investigate the moderating effect of the sleep quality and education level on the relationship between depression and suicidal ideations among middle-aged parents of adolescent children. In addition, we examined the research model by exploring the moderated moderation effect of education level on the relationship between depression, sleep quality, and suicidal ideations.

First, the study revealed that middle-aged adults showed that the suicidal ideations based on the SIQ were 8.13 ± 19.56 points. This score was lower than in Reynolds’s study (1991; 11.43 ± 14.60) [37] and 10.19 points [38] in a study targeting university students (average age of 21 years old). This is because it is believed that the number of suicide ideations among middle-aged adults, who are relatively less impulsive than adolescents, was measured lower [39]. The depression score (BDI-II) was 6.34 ± 6.45 points, which was lower than 8.36 ± 6.59 points and 9.36 ± 7.72 points, respectively, in the studies of Sung et al. [40] and Shin [41] targeting middle-aged women in Korea. This result may be due to the fact that the study included male adults compared to the existing studies that only studied middle-aged women. However, the result was similar to the 6.04 ± 7.53 scores in a study of middle-aged Italians [42], so it can be inferred that middle-aged adults are globally less aware of depression or are less affected by depression than older adults or university students. In this study, the sleep quality (PSQI) was 4.24 ± 2.57 points, which was lower than the findings of Buysse et al. [43] in middle-aged adults in Pittsburgh, PA in the United States (6.3 ± 3.4 points). Compared to 11.58 ± 6.99 points in other age groups in Korea [44], the participants in this study had relatively good sleep quality. This differs from previous reports that found that adults in their thirties and fifties have shorter sleep times when compared to other age groups and that middle-aged parents with children under the age of 18 have shorter sleep times than middle-aged parents with children in other age groups [16,18]. This is because factors other than sleep time act in evaluating the sleep quality, so children of different age groups or adults of different age groups should be used for comparative research using the same tool in the future. It is necessary to conduct a follow-up study targeting middle-aged parents of children of different age groups.

Second, a correlational analysis confirmed that depression and sleep quality had a positive correlation with suicidal ideations. This is consistent with the results of previous studies that found that the more severe the depression, the more the suicidal ideations increased [16,45]. In addition, a multiple regression analysis confirmed that depression had a significant effect on suicidal ideations. This result was consistent with a study that found that the severity of depression was associated with more suicidal ideations in middle-aged men [46] and in the elderly [45,47,48]. This result also implies that, because suicidal ideation is the main variable of suicide risk, a group with high levels of depression should be considered a target group for suicide prevention. According to a study by Choi et al. [49], ideations of suicide were 3.5 times more frequent in adults who had experience with depression than those who did not. In addition, there was no difference in the presence or absence of suicidal ideations among those who had no experience with depression [49]. As such, suicidal ideations differed depending on the presence or absence of depression, so a screening test for depression should be considered when planning an education program to prevent suicide. In addition, suicidal ideations also showed a positive correlation with sleep quality, which supported the results of Tae et al. [50]. This is similar to the results of previous studies of adolescents and adults, in which shorter sleep times resulted in more frequent suicidal ideations [51,52]. Nursing interventions can have the effect of lowering suicidal ideations, which can lower the depression level and improve the sleep quality. Specifically, evidence-based programs to alleviate depression, such as art therapy, aerobics, stretching, laughter therapy, and aroma massage [53,54], and those to improve sleep quality, such as ear pressure therapy, foot massage, and aroma therapy [55], should be recommended for middle-aged adults. Third, there was a simple moderating effect of the sleep quality and education level on the relationship between depression and suicidal ideations. There was no previous study targeting general adults, but this result was consistent with the finding that depressed adults who reported severe insomnia had more suicide attempts than the group with only depression [47,56]. In addition, this is consistent with the results showing that sleep disturbances increase suicidal ideations in depressed patients, and the influence of sleep disturbances on suicidal ideations decrease when the level of depression is low [57]. These results confirmed that sleep quality has a moderating effect on the effect of depression on suicidal ideations. Therefore, when planning suicide prevention programs for middle-aged adults, it is important to add the context of improving the sleep quality as well as depression management. Moreover, the education level had a moderating effect on the relationship between depression and suicidal ideations. Therefore, it would be more beneficial to focus on managing depression and sleep quality, especially if education for mental health is provided for middle-aged adults with higher education. Studies have not yet reported on the moderating effect of education levels on the relationship between depression and suicidal ideations, but the results of this study support the finding that subjects with low education levels are more likely to experience suicidal ideations [17,45,46,58]. This result implies that it is beneficial to change the content of suicide prevention programs, including depression and sleep quality, according to the education level. Although it cannot be said that classification according to the education level is necessarily consistent with the level of literacy, considering the report that the higher the education level, the higher the level of literacy [59,60], it is important to consider participants’ education level when developing individualized suicide prevention programs [61]. Moreover, the lower the level of health literacy level, the more negative and passive the attitudes are toward health behaviors [62]; therefore, it is necessary to develop and utilize various media to enhance the understanding of the importance of managing depression and sleep quality according to the target audience [63,64]. In this way, if the subject’s health literacy is investigated in advance and an educational program that can improve depression and the sleep quality is developed accordingly, a greater effect can be expected in reducing suicidal ideations. Fourth, the study confirmed that the moderated moderation effect of the education level was related to the effect of sleep quality, which showed a moderating effect on the effect of depression on suicidal ideations. According to a study conducted by Sami et al. [25] on adolescents, when middle-aged parents of adolescent children have low levels of education, the sleep quality is lower than in middle-aged parents with high levels of education. As such, existing studies have reported the relationship between depression, sleep, and suicidal ideations, but the effect of the education level on these relationships has been overlooked in studies targeting middle-aged adults, as well as older adults [16,50,64]. However, in this study, it was found that the effect of depression on suicidal ideations was greater when the sleep quality was poor for those with a higher and less than university graduation. In addition, a simple slope effect test of the moderated moderation effect of the education level showed that, in high school graduation groups, the effect of depression on suicidal ideations was not statistically significant. This suggests that, even in the higher education level group of depressed people, sleep hygiene education should be better during suicide prevention education. Moreover, in the lower education level group, it is necessary to examine the moderating effects of variables other than sleep quality between depression and suicidal ideations. In summary, the influence of depression on suicidal ideations through improved sleep quality can be reduced by developing and utilizing educational methods and intervention programs for improving sleep hygiene when taking into consideration the subject’s education level. Therefore, it might be more beneficial for middle-aged parents who have depression or have been diagnosed at a hospital to be given education level assessments in developing suicide prevention programs. To the best of our knowledge, this study is one of the few to examine the moderating effects of sleep quality and education level on the relationship between depression and suicidal ideations among middle-aged parents who have adolescent children. Further studies should be conducted to extend the implications of the results of this study and to confirm the effects of depression and suicidal ideations in a larger sample from different regions. Despite the importance of the findings of this study, several limitations should be discussed. First, other influencing factors identified in previous studies as variables related to depression and suicidal ideations—such as economic status and the body mass index—should be examined. Second, the effects of suicidal ideations and sleep quality between depressed and nondepressed groups and their mothers and fathers should be identified. Third, our study participants were recruited via convenience sampling in two schools, and 91.6% of the participants were women. The sleep quality, depression, and suicidal ideations were related to gender, so there was possibility of a limited generalizability of this study’s results. Therefore, a larger sample, especially male, in different regions should be collected. Fourth, we used a cross-sectional design and, thus, could not establish a causal relationship between the study variables. Fifth, the sleep quality was assessed based on the participants’ recollection of their past experiences; thus, a memory bias cannot be excluded. Therefore, we recommend the use of objective data such as actigraphy for the sleep quality. Sixth, since the education level of the subjects does not reflect the health literacy, it is necessary to be careful in interpreting the results.

## 5. Conclusions

This study proved not only the simple moderating effect of the sleep quality and education level but, also, the moderated moderation effect of the education level on the relationship between depression, sleep quality, and suicidal ideations. The results imply that there is a need for research and education that focuses on individualized educational contents on depression and the sleep quality, with delivery methods according to the education level, in order to prevent suicide among middle-aged adults. Adolescence is a sensitive period that is highly influenced by middle-aged parents; therefore, the mental health management of middle-aged parents is necessary, but also, the mental health management of adolescent children is needed. Therefore, it is beneficial to investigate and manage middle-aged parents of adolescents at the school level, and to prevent suicide, a screening test for depression, as well as a test for sleep hygiene, is necessary. In addition, it is beneficial to develop content according to the target group’s education level and to reinforce sleep hygiene education in target groups with lower levels of education.

## Figures and Tables

**Table 1:** Characteristics of the participants (*n* = 178).

Variables		Mean (SD) or *n* (%)
Gender	Male	15 (8.4)
	Female	163 (91.6)
Age(years)		46.28 (±3.21)
Education	<University	114 (64.4)
	≥University	63 (35.6)

SD, Standard deviation.

**Table 2:** Pearson correlations among the suicidal ideations, depression, and sleep quality (*n* = 178).

	Suicidal Ideations	Depression	Sleep Quality	Age
Suicidal ideations	1			
Depression	0.59 (<0.001)	1		
Sleep quality	0.34 (<0.001)	0.37 (<0.001)	1	
Age	0.15 (0.046)	0.08 (0.276)	−0.07 (0.382)	1
Mean	8.13	6.34	4.24	46.28
SD	19.56	6.45	2.57	3.21

SD, Standard deviation.

**Table 3:** Moderated moderation effect of sleep quality and education level between depression and suicidal ideations.

Effect *, Variable	Model 1 ^a^			Model 2 ^b^			Model 3 ^c^		
	B	se	*p*	B	se	*p*	B	se	*p*
depression	0.92 ***	0.18	<0.001	−0.52	0.42	0.220	0.78	0.52	0.134
sleep quality	0.87 *	0.42	0.040	−0.55	0.58	0.347	0.66	0.70	0.362
Education (University)	3.19	2.07	0.126	−4.07	2.87	0.159	9.57	5.28	0.072
interaction effect									
Depression × PSQI				0.21 *	0.07	0.003	−0.03	0.09	0.736
Depression × Education (University)				1.15 *	0.37	0.002	−1.80 *	0.82	0.029
PSQI × Education (University)							−2.93 *	1.17	0.013
Depression × PSQI × Education (University)							0.53 ***	0.14	<0.001
R^2^ (⊿R^2^) F(*p*)	0.21, *p* < 0.001 10.41 (<0.001)			0.30 (0.09), *p* < 0.001 11.08 (<0.001)			0.36 (0.07), *p* = 0.001 11.04 (<0.001)		

^a^ Model 1: the model that controls gender and age, unstandardized regression coefficient (B), standard error (se), and coefficient of determination(R^2^). ^b^ Model 2: the model with a moderating effect on the sleep quality and education level. ^c^ Model 3: the model with a moderated moderation effect on the education level. * *p* < 0.05 and *** *p* < 0.001.
